# Daily steps and healthcare costs in Japanese communities

**DOI:** 10.1038/s41598-021-94553-2

**Published:** 2021-07-23

**Authors:** Shohei Okamoto, Kazuki Kamimura, Kenichi Shiraishi, Kazuto Sumita, Kohei Komamura, Akiko Tsukao, Shoko Chijiki, Shinya Kuno

**Affiliations:** 1 Research Team for Social Participation and Community Health, Tokyo Metropolitan Institute of Gerontology, 35-2 Sakae-cho, Itabashi-ku, Tokyo, 173-0015 Japan; 2 Institute for Global Health Policy Research, National Centre for Global Health and Medicine, Tokyo, Japan; 3 Hirao School of Management, Konan University , Hyogo, Japan; 4 Department of Social Welfare, Gunma University of Health and Welfare , Gunma, Japan; 5 Department of International Economics, Toyo University , Tokyo, Japan; 6 Faculty of Economics, Keio University , Tokyo, Japan; 7 Tsukuba Wellness Research, Inc., Chiba, Japan; 8 R&D Center for Smart Wellness City Policies, University of Tsukuba , Ibaraki, Japan

**Keywords:** Health care, Health care economics, Health policy, Health services, Public health

## Abstract

Physical inactivity is a pandemic that requires intensive, usually costly efforts for risk reduction of related chronic diseases. Nevertheless, it is challenging to determine the effectiveness of physical activity in healthcare cost reduction based on existing literature. Therefore, this study aimed to investigate the impact of physical activity (daily steps) on healthcare costs utilising the data retrieved from a health promotion project (*the e-wellness Project*, held in three municipalities in Japan). Evaluating the effects of daily steps, measured by pedometers, on healthcare costs by a quasi-experimental approach among participants aged 40–75 years (about 4000 person-years of observation, between 2009 and 2013), we found that a one-step-increase in the annual average daily step reduced outpatient healthcare costs by 16.26 JPY (≒ 0.11 GBD) in the short run. Based on the assumption of a dynamic relationship between the health statuses in multiple years, the long-run effects of daily steps on healthcare costs were estimated at 28.24 JPY (≒ 0.20 GBD). We determined the health benefits of walking in a sample of middle-aged and older Japanese adults by our findings that an increase in step counts reduced healthcare costs.

## Introduction

Physical inactivity, a pandemic that requires global efforts^1,2^, contributes to a significant increase in the disease burden of non-communicable diseases. Thus, being active is a well-known healthy behaviour that is effective in preventing non-communicable diseases^3–5^, depression^6^, and dementia^7^. In Japan, physical inactivity is one of the major preventable risk factors for mortality in adults^8^. The Japan age-standardised prevalence of physical inactivity, defined as attaining less than 150 minutes of moderate-intensity or less than 75 minutes of vigorous-intensity physical activity per week, or equivalent, is 33.8% and 37% in men and women, respectively. These are higher than the global prevalence of 23.4% and 31.7% in men and women, respectively^2,9^.

In addition, physical inactivity is associated with an economic burden. A previous study estimated the social cost of physical inactivity, which comprises direct healthcare costs, disability-adjusted life-years for related diseases, and productivity losses^10^. The authors reported that physical inactivity generated a substantial cost of international $53.8 billion, which is mostly paid by the public sector. The finance of healthcare costs, particularly by insurance premiums or taxes, implies that healthy individuals bear the burden arising from inactive people since the contribution of physical activity is not linked to healthcare systems. Nevertheless, it is nearly impossible to impose direct taxes on inactive individuals unlike the tobacco and alcohol excise taxes^11^.

Thus, interventions (e.g. incentive mechanisms) seem feasible for changing people’s lifestyles from inactive to active to reduce both financial and non-financial burdens. However, the effectiveness of such intervention schemes is not explicitly known^12^.

Previous studies investigated the relationship between physical activity and healthcare usages/costs. Sari^13^ reviewed the literature on physical activity and healthcare utilisation in older adults and suggested that physical activity reduces the utilisation of healthcare services. However, significant variations occurred in samples, methods, and estimated effects of physical activity on healthcare services usage between studies, which makes it difficult to generalise the effects.

Other studies that investigated the association between physical activity and healthcare costs, suggest that undergoing sufficient levels of physical activity are associated with decreased healthcare costs^14–20^. These studies, however, lacked objective measurements of physical activity or healthcare records. In addition, the trajectory of physical activity during the follow-up was not considered. Most importantly, they failed to address potential endogeneity owing to a lack of statistical causal inference, which could lead to a biased estimation of the health effects of physical activity.

Therefore, this study aimed to investigate the impacts of physical activity (daily steps) on healthcare costs, to contribute to the existing literature in these two main ways. First, we utilised *the e-wellness project* data that were continuously recorded for at most 5 years, and which include objective measurements of both daily step and healthcare costs. Second, we adopted a quasi-experimental approach to assess the impact of daily steps on healthcare costs, dealing with the endogenous relationship between daily steps and health.

## Methods

### Data

Our sample analysed in this study comprised the participants of *the e-wellness project*, an individualised health promotion programme implemented by the University of Tsukuba in Japan. The project was conducted in three cities of Fukushima, Niigata, and Gifu Prefectures from April 2009 to March 2013. The participants in the e-wellness project were volunteer residents aged 40–74 years old, corresponding to the targeted population of Japan’s public health policy for preventive healthcare (i.e. Specific Health Check-ups and Specific Health Guidance). Individuals who were unable to exercise by a physician’s instruction were not eligible to participate.

The main focus of the project was to improve the health habits of the participants on walking, muscle training, and dietary habits. Face-to-face or telephonic supports and instructions were provided for each participant. These included exercise classes and individualised bits of advice on how to increase the daily steps, contents/intensity/frequency of muscle training and other exercise, and a nutrition guide with a plan on how to manage one’s body weight.

This study was approved by the Institutional Review Board of the University of Tsukuba (Tai24-27). All participants provided written informed consent, and all methods were performed in accordance with the relevant guidelines and regulations.

### Healthcare costs

We obtained data on healthcare costs of participants from the National Health Insurance (NHI) claims records from April 2009 to March 2013. Healthcare costs data included the costs for both outpatient and inpatient care. Healthcare records for a total of 4,003 person-years were obtained for analysis.

To address the skewed distribution of healthcare cost data, we pooled the data on all residents in the three cities covered by the NHI during the aforementioned period (N = 748,905 person-years) and excluded those with the top 1% costs. Consequently, 19 and 12 person-years’ data of participants for outpatient and inpatient services, respectively, were excluded from our analyses.

### Daily step data

The number of daily steps was measured by a pedometer (model: H-J730IT and J-J740IT, Omron Healthcare Co., Ltd.), which was assigned to each participant in the e-wellness project. Each participant was asked to attach the pedometer to the waist and wear it all day long, from the time they wake up until they go to bed. There was no difference in step counts between H-J730IT and J-J740IT pedometers. Pedometers used in this project met the following reliability and validity criteria, which was confirmed throughout meetings between the manufacturer and researchers: (1) less than 5% measurement errors on average compared with the actual step counts and (2) less than 5% measurement errors on average compared to a trunk-mounted pedometer with a three-axis acceleration sensor.

The monthly average daily step was recorded and transformed into the annual average to make it comparable with the healthcare costs. Since the e-wellness participants uploaded their walking data by themselves, there were missing data. On average, the data for about 9 months per year were available. Therefore, we calculated the annual average daily step of the participants from the available data even when the recorded period of daily step in a year was less than 12 months.

### Identification strategy

In the current paper, we evaluated the impacts of daily steps on healthcare costs. However, there were methodological challenges to estimating the impacts that existing studies have paid little attention to. The potential endogeneity which violates an assumption of the consistency of the ordinary least squares (OLS) regression owing to the unobserved heterogeneity and reversed causality, is problematic. In this study, we estimated the following dynamic panel model (1):

1 $${HC}_{i, t}=\alpha {HC}_{i, t-1}+\beta {Step}_{i, t}+\gamma {Age}_{i, t}+{\varepsilon }_{i, t}$$

$${\varepsilon }_{i, t}={\mu }_{i}+{\nu }_{i, t}$$

where ${HC}_{i, t}$ denotes healthcare costs*,* ${Step}_{i, t}$ is the annual average count of daily steps, and ${Age}_{i, t}$ refers to the age of an individual *i* in year *t.* ${\varepsilon }_{i, t}$ is the error term, which comprises the fixed effects ${\mu }_{i}$ and the idiosyncratic shocks ${\nu }_{i, t}$. It is a natural assumption that the present health status or healthcare utilisation is affected by the past one, as shown in Eq. (1). However, the estimation of OLS using Eq. (1) causes endogeneity owing to the correlation between healthcare costs in the previous year and the fixed effects, and the correlation remained even after individual fixed effects were removed^21^.

Thus, removing the fixed effects and finding the instrument ${HC}_{i, t-1}$ with variables uncorrelated with the fixed effects is necessary to perform causal inference. Therefore, we dealt with the endogeneity by a quasi-experimental approach based on the system Generalized Method of Moments (GMM) developed by Arellano and Bover^22^ and Blundell and Bond^23^. System GMM uses the lags in the levels of the first-difference variables as the internal instruments to ${HC}_{i, t-1}$ and ${Step}_{i, t}$, which are considered to be endogenous to idiosyncratic shocks.

To test the validity of the instruments, we used the Hansen test for overidentification and the Arellano–Bond test for autocorrelation. The Hansen test assesses the validity of the instruments by testing the null hypothesis that all instruments are not correlated with the idiosyncratic shocks based on the robust variance–covariance estimator. The Arellano–Bond test assesses the serial correlation of the idiosyncratic shocks that potentially makes the instruments endogenous to the idiosyncratic shocks. The second-order correlation [AR(2)] in the differences (i.e. the correlation between ${\upsilon }_{i, t-1}$ in ${\Delta \nu }_{i, t}$ and the ${\upsilon }_{i, t-2}$ in ${\Delta \nu }_{i, t-2}$) is tested as the first-order correlation is naturally expected by the shared ${\upsilon }_{i, t-1}$.

In Eq. (1), $\beta$ measures the immediate impact on healthcare costs within the year, which does not consider its dynamic impacts. Therefore, we estimated the long-term effects of daily steps on healthcare costs by the coefficient of daily steps divided by 1 minus the lagged coefficient of the dependent variable^24^.

Additionally, we controlled for age, city, and time fixed-effects to consider differences in factors that potentially affect healthcare costs across the survey years and municipalities (e.g. changes in the number of hospitals and different epidemic situations such as flu).

All the analyses are performed in Stata, version 16.1. System GMM models were estimated using the Stata command *xtabond2*^25^.

## Results

### Effects of daily steps

**Figure 1:**
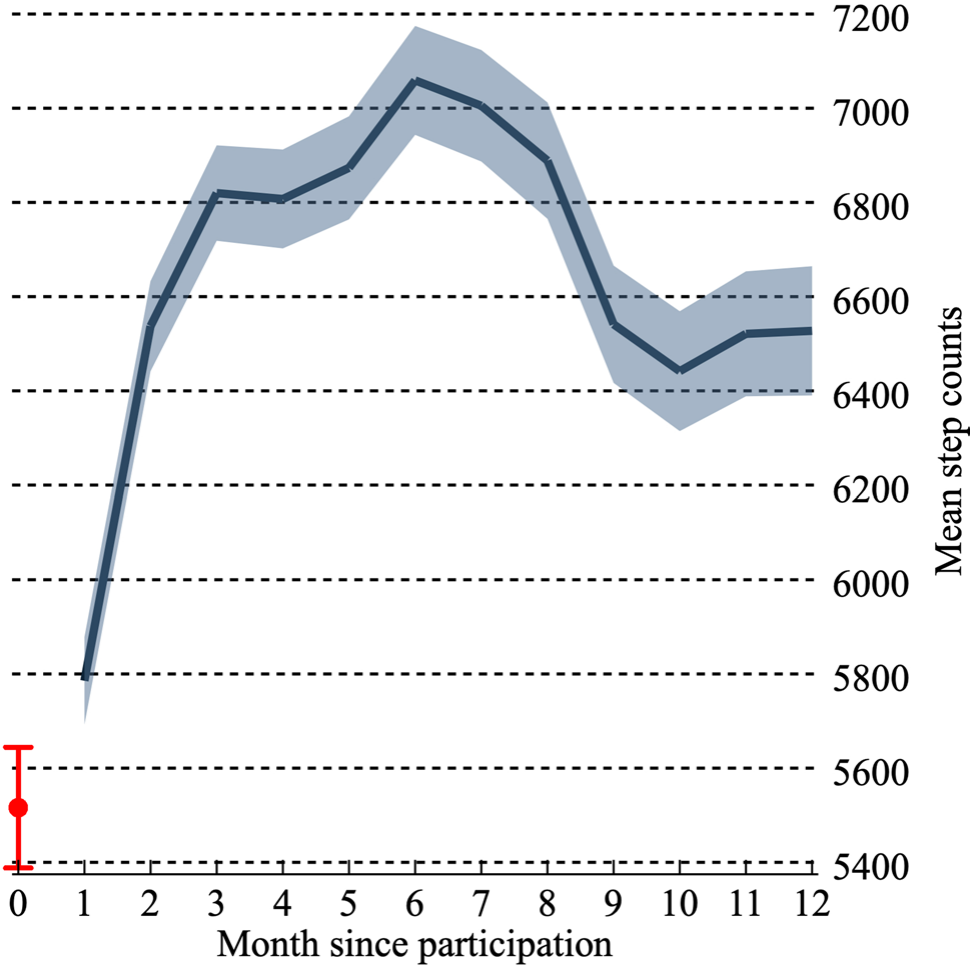
Mean step counts after participation in the e-wellness project. The red marker with the error bar represents mean step counts and the 95% confidence interval among the participants at the baseline. Navy lines represent mean step counts after participation in the e-wellness project, with shaded areas showing the 95% confidence intervals among the participants. Sample sizes for each month step counts range from 1,115 to 1,536. This figure was created by the Stata software, version 16.1.

Table 1 shows a summary of the descriptive statistical analysis. The annual average outpatient and inpatient costs were 240,606 JPY (≒ 1690 GBD) and 43,361 JPY (≒ 304 GBD), respectively. Men were older than women, and on average, spent more on healthcare costs than women, both for outpatient and inpatient services. The average age of the participants was 65.8 years, and 76% of the participants were women. The average annual step counts throughout the follow-up were 6,871, and these gradually increased after the participation of individuals in the health promotion project; then they stabilised, 9 months after an average increase of about 1000 steps (Fig. 1).

**Table 1:** Summary statistics.

Variable^a^	(i) Whole		(ii) Male		(iii) Female		(ii)–(iii)^b^
	Mean	SD	Mean	SD	Mean	SD	
Outpatient costs (JPY)^c^	240,606	205,198	271,217	225,182	231,180	197,726	***
Inpatient costs (JPY)	43,361	226,507	69,346	284,858	35,366	204,639	***
Daily steps (annual ave.)	6871	2935	7496	3471	6679	2720	***
Age	65.84	5.60	67.118	5.584	65.448	5.552	***
Male (= 1)	0.24	0.42	1.000	0.000	0.000	0.000	
City A (= 1)	0.33	0.47	0.318	0.466	0.339	0.474	
City B (= 1)	0.44	0.50	0.433	0.496	0.438	0.496	
City C (= 1)	0.23	0.42	0.249	0.433	0.222	0.416	
2009(= 1)	0.15	0.36	0.131	0.338	0.158	0.364	^##^
2010(= 1)	0.19	0.39	0.186	0.389	0.191	0.393	
2011(= 1)	0.20	0.40	0.205	0.404	0.202	0.401	
2012(= 1)	0.23	0.42	0.232	0.423	0.226	0.418	
2013(= 1)	0.23	0.42	0.246	0.431	0.223	0.416	
N. of observations	3984		938		3046		

*JPY* Japanese Yen, *Annual ave*. Annual average, *SD* standard deviation.

^a^(= 1) indicates dummy variable.

^b^***,** indicate the positive results of a t-test of equal means between two samples at the 1% and 5% significance levels, respectively. ###,## indicate the negative results of the t-test at the 1% and 5% significance levels, respectively. We use Welch’s method to test the difference of averages under the hypothesis of heteroskedasticity.

^c^The sample size for inpatient costs is different (whole: n = 3991, male: n = 939, female: n = 3052).

Table 2 represents the estimation results of the fixed-effects OLS regression and system GMM. The validity of the model by system GMM was confirmed by the Hansen test and Arellano-Bond test for second-order correlation [AR(2)]. We found that daily steps were associated with reduced healthcare costs for outpatient services in both models. These effects were larger in the dynamic panel model, which dealt with the potential endogeneity (OLS, β: − 14.00, robust standard error [SE]: 3.08 vs system GMM, β: − 16.26, SE: 6.51). The long-run effects of daily steps on outpatient costs were estimated to be − 28.24 (SE: 11.45).

**Table 2:** Effects of daily step on healthcare costs: outpatient services.

Variable	1. Fixed-effects OLS			2. System GMM		
	Whole	Male	Female	Whole	Male	Female
Daily steps	− 14.00*** (3.08)	− 8.88 (4.96)	− 16.96*** (3.89)	− 16.26** (6.51)	− 6.65 (9.55)	− 23.18*** (7.84)
Daily steps: long run effect				− 28.24** (11.45)	− 11.85 (17.00)	− 42.39*** (14.37)
Outpatient costs (JPY) (t − 1)				0.42*** (0.05)	0.44*** (0.12)	0.45*** (0.07)
Age	15,366*** (5508)	7992 (13,400)	18,186*** (5908)	4834*** (872.7)	3108 (1841)	5048*** (1077)
Male (= 1)				30,181*** (11,318)		
Individual fixed effects	Yes	Yes	Yes	Yes	Yes	Yes
Year fixed effects	Yes	Yes	Yes	Yes	Yes	Yes
City fixed effects				Yes	Yes	Yes
Constant	− 673,483 (372,475)	− 175,202 (922,080)	− 852,311** (397,426)	− 48,768 (66,706)	21,508 (146,504)	− 27,905 (75,059)
Number of observations	3984	938	3046	2430	565	1865
Number of individuals	1532	368	1164	1074	259	815
1st stage specification tests						
Arellano-bond: AR(2) z				− 0.497 [0.619]	1.014 [0.310]	− 1.059 [0.290]
Hansen J				15.46 [0.419]	13.21 [0.586]	15.42 [0.422]
Hansen J p value						
Number of Instruments				26	25	25

Robust standard errors in parentheses. ***p < 0.01, **p < 0.05; p values in brackets.

The long-run effects of daily step on healthcare costs are calculated by the coefficient of daily steps divided by 1 minus the lagged coefficient of the dependent variable.

*OLS* ordinary least squares, *GMM* generalized method of moments.

In addition, we observed a significant difference between sexes. Daily steps significantly reduced healthcare costs in women (OLS, β: − 16.96, SE: 3.89 vs system GMM, β: − 23.18, SE: 7.84), while the effects of daily steps on healthcare costs were not significant in men. The long-run effects of daily steps on outpatient costs were estimated to be − 42.39 (SE: 14.37) in women, which is greater than those for men only and those for the whole sample; although it is possible that the insignificant results obtained for men are due to the small sample size.

Table 3 shows the results for inpatient costs. The validity of the model by system GMM was confirmed by two tests (see “Methods” section for details of the tests). However, no impact of daily steps on inpatient costs was observed after dealing with the potential endogeneity by system GMM, although the association by OLS was found to be significant in the whole sample (whole, β: − 17.25, SE: 6.03) and in women (β: − 18.60, SE: 6.37).

**Table 3:** Effects of daily step on healthcare costs: inpatient services.

Variable	1. Fixed-effects OLS			2. System GMM		
	Whole	Male	Female	Whole	Male	Female
Daily steps	− 17.25*** (6.03)	− 13.83 (12.36)	− 18.60*** (6.37)	0.64 (13.47)	1.82 (24.39)	1.89 (9.82)
Daily steps: long run effect				0.72 (15.31)	2.15 (28.96)	2.18 (11.33)
Inpatient costs (JPY) (t − 1)				0.12 (0.07)	0.15 (0.10)	0.13** (0.06)
Age	− 108.7 (11,173)	71,186** (35,674)	− 21,429** (9,352)	2418*** (772.9)	4405 (2294)	1426 (805.8)
Male (= 1)				31,695** (15,511)		
Individual fixed effects	Yes	Yes	Yes	Yes	Yes	Yes
Year fixed effects	Yes	Yes	Yes	Yes	Yes	Yes
City fixed effects				Yes	Yes	Yes
Constant	181,956 (748,091)	− 4.652e + 06* (2.416e + 06)	1.594e + 06** (633,703)	− 131,343 (101,732)	− 240,541 (279,446)	− 85,415 (84,384)
Number of observations	3991	939	3052	2434	565	1869
Number of individuals	1534	368	1166	1069	256	813
1st stage specification tests						
Arellano-bond: AR(2) z				0.674 [0.500]	0.260 [0.795]	0.278 [0.781]
Hansen J				16.16 [0.372]	12.99 [0.603]	15.03 [0.450]
Number of Instruments				26	25	25

Robust standard errors in parentheses. ***p < 0.01, **p < 0.05; p values in brackets.

The long-run effects of daily step on healthcare costs are calculated by the coefficient of daily steps divided by 1 minus the lagged coefficient of the dependent variable.

*OLS* ordinary least squares, *GMM* generalized method of moments.

### Robustness checks

In our main analyses, we found that daily steps reduced outpatient costs. To test the robustness of our findings, we further conducted three additional analyses as follows. First, to account for the potential endogeneity, we treated daily steps, $Ste{p}_{i,t}$ (annual average count of daily steps), as an endogenous variable and estimated the model by system GMM only, using the internal instruments. However, Bazzi and Clemens^26^ suggested that system GMM estimator based on internal instruments has a potential weak instrument problem. Therefore, we estimated the models by including additional external instruments of daily steps for the first robustness checks, assuming that the internal GMM instruments are potentially weak, and the external instruments are likely strong (Appendix A). We confirmed that the results remained unchanged.

Second, in Appendix B, we performed additional analyses using multiple imputation methods since we did not have sufficient information to control for potential confounders owing to the limited access to individual-level data. Specifically, we added the information on metabolic syndrome as a control variable by creating a dummy variable, which takes a value of 1 if the category was either metabolic or pre-metabolic syndrome, and 0 if otherwise. However, about a quarter of the information on metabolic syndrome was missing because of those who did not undergo a health check-up. Therefore, we dealt with the missing metabolic syndrome data by the multiple imputation method. We included dummy variables for age, sex, medical expenses for outpatient/inpatient services, annual average daily step counts, residential city, and year, as independent variables in the imputation model, analysed by logistic regression. We confirm that the results were still robust.

Third, we augmented the information on an economic factor (i.e. personal income), which is an important determinant of healthcare spending that allows individuals to allocate more resources to health^27^, by using a data augmentation method to guarantee the robustness of our findings against a potential omitted-variable bias (Appendix C). Consequently, we confirm that our findings remained unchanged even after controlling for the economic factor.

## Discussion

### Contributions of this paper

While previous studies suggested that physical activity can be effective to reduce healthcare costs, the evidence was not sufficiently robust because of three reasons, at least. First, previous studies lacked an objective measurement of physical activity or healthcare costs data, which led to a hypothetical study on the relationship. Second, a longitudinal relationship based on panel data, in which both physical activity and healthcare costs data were measured during the follow up, was rarely analysed. Third, previous studies did not properly assess the effects of physical activity on healthcare costs owing to their insufficient approach to deal with potential endogeneity.

Therefore, our study estimated the impacts of daily steps measured using a pedometer on healthcare costs, based on the panel data of *the e-wellness project*. Moreover, we assessed the effect of daily steps on healthcare costs using an instrumental variable approach, which enabled us to deal with potential endogeneity. Consequently, we found that the one-step-increase in the annual average daily step (that is, a 365-step increase in total, a year) reduced outpatient healthcare costs by 16.26 JPY (≒ 0.11 GBD) in the short run (i.e. within a year). Based on the assumption that there was a dynamic relationship between the health statuses in multiple years, the long-run effects of daily steps on healthcare costs were estimated to be 28.24 JPY (≒ 0.20 GBD).

### Comparison with previous studies

While it is difficult to compare the findings in this study with other studies due to different measures of physical activity, some previous studies in Japan speculated on the reduction in healthcare costs due to walking. Yoshizawa, et al.^17^ found that outpatients’ healthcare costs per year in a control group were higher than those in the intervention group by 361.9 USD (≒ 278 GBD) although they failed to address endogeneity bias. The step counts in the intervention group gradually increased by 1,500 steps and stabilised after 15 months, and the effect of one step increase was estimated to be a decreased by about 0.24 USD (≒ 0.18 GBD, 26.3 JPY) in outpatient costs. Although another study^15^ lacked causal inference and objective step counts data, they reported that the outpatient healthcare costs per month for those walking more than an hour and those walking between thirty minutes and an hour a day were £60.9 and £69.5, respectively. For a rough comparison, assuming that a 10 min-walk is equivalent to 1,000 step counts (that is, an hour vs 0.75 h were ≒ 6,000 vs 4,500 steps) and individuals had the endpoint or a median point step counts, the increase in one step count is estimated to save £ 0.007 (≒ 9.8 JPY). Our study, which included both an objective measurement of step counts and causal inference, reported a saving of 16.26 JPY (≒ 0.11 GBD) for an increase of one step count, and this value falls within those reported in previous studies.

### Sex differences

We observed heterogeneous impacts of step counts on healthcare costs by sex. The health effects of daily steps were found to be significant only in women. This might be owing to sex differences in sample sizes, that is, more women participated in the e-wellness project than men. A previous study suggested that women were more likely to adopt preventive health behaviours than men^28^, which may partly explain the higher participation rate of women than men in the e-wellness project.

### Limitations

While our study has unique strengths, there are several limitations. First, we can interpret our findings to be the improvements in health condition in relation to physical and mental health, by inferring from previous findings^3–7^. However, since the onset of diseases was not followed up in our study, we were unable to evaluate the detailed mechanism of the health effects of physical activity and only assessed the overall effects of daily walking on health.

Second, we did not have sufficient information to control for potential confounders. Controlling for healthcare costs in the previous year by a dynamic panel data model may deal with the issue of unmeasured confounders. In addition, we checked the robustness of our findings by multiple imputation method (Appendix B) and by augmenting income data (Appendix C). However, we still need to be cautious in our interpretation owing to the lack of sufficient control variables.

Third, our findings may not be applicable to the general population since the participants of the e-wellness project were those who voluntarily chose to participate in a health intervention. Moreover, potential differences in pedometer wear time or compliance could impact the accuracy of daily step counts. Thus, a further investigation to improve the external validity as well as the accuracy of data about physical activity is required. Furthermore, cities where the project took place were not metropolitan cities, where public transportation is less convenient than metropolitan areas. As the differences between urban and rural areas in physical activity, the physical environment, and the impacts of psychosocial factors exist^29^, further studies should reveal the potential heterogeneity across regions to obtain generalisable results.

Fourth, the follow-up period of our study, at most 5 years, might be insufficient to evaluate the long-term effects of physical activity on healthcare costs. In this study, we observed the reduction in outpatient costs, not in inpatient costs, which could be explained by the short follow-up period, since the impacts of walking on the onset of a severe illness may not appear immediately. Ideally, a follow-up of individuals until the time of death would be helpful to assess the long-term health effects of the intervention. If the onset of a disease is just delayed by an intervention but is observed eventually, it is difficult to conclude that the intervention is effective in terms of reducing healthcare costs. While it is valuable in improving individuals’ health, the budget constraints restrict the government from spending enormous costs in implementing such programmes. Thus, the cost-effectiveness of the intervention, in the long run, should be evaluated to improve the health policy implications. Although the current study was unable to consider the costs of the project and the quality aspects of life, further studies should investigate the long-term cost-effectiveness of the intervention.

The current health promotion in Japan was initiated as *Health Japan 21 (the second term),* which set concrete goals for each health outcome. The average daily steps of Japanese people are steady but are aimed to be increased by more than 1,000 steps in a few years^30^. The Ministry of Health, Labour and Welfare in Japan has approached this goal by advertising the ‘+10 (plus ten)’ goal, which encourages people to spend 10 more minutes a day on physical activity^31^. This approach has been described as the weakest intervention besides the *do nothing or simply monitor*^32^. A stronger intervention than advertisement may be necessary to achieve the goal after considering its justifications and cost-effectiveness in the future.

In conclusion, we determined the health benefits of walking in a sample of middle-aged and older Japanese individuals in three cities using objective data of daily step counts and healthcare costs, and found that an increase in step counts reduced healthcare costs.

## Supplementary Information

Supplementary Information 1.
