# Supplementary material for: Daily steps and healthcare costs in Japanese communities
Source: Sci Rep. 2021 Jul 23;11:15095. doi: 10.1038/s41598-021-94553-2 (PMC8302729; doi:10.1038/s41598-021-94553-2)
Supplement: Supplementary file 1 — Supplementary Information 1. [file 41598_2021_94553_MOESM1_ESM.pdf]

Supplementary Material for
Daily steps and healthcare costs in Japanese communities

Shohei Okamoto[1, 2], Kazuki Kamimura[3], Kenichi Shiraishi[4], Kazuto Sumita[5], Kohei Komamura[6], Akiko Tsukao[7], Shoko Chijiki[7], Shinya Kuno[8]

1: Research Team for Social Participation and Community Health, Tokyo Metropolitan Institute of Gerontology, Tokyo, Japan
2: Institute for Global Health Policy Research, National Center for Global Health and Medicine, Tokyo, Japan
3: Hirao School of Management, Konan University, Hyogo, Japan
4: Department of Social Welfare, Gunma University of Health and Welfare, Gunma, Japan
5: Department of International Economics, Toyo University, Tokyo, Japan
6: Faculty of Economic, Keio University, Tokyo, Japan
7: Tsukuba Wellness Research, Inc., Chiba, Japan
8: R&D Center for Smart Wellness City Policies, University of Tsukuba, Ibaraki, Japan

Corresponding author: Shohei Okamoto
Research Team for Social Participation and Community Health, Tokyo Metropolitan Institute of Gerontology 35-2 Sakae-cho, Itabashi-ku, Tokyo, Japan 173-0015
E-mail: sokamoto@tmig.or.jp

Contents

A. Robustness check for an instrumental variable approach

B. Multiple imputation

C. Data augmentation of personal income from panel survey

References for Supplementary Material

A. Robustness check for an instrumental variable approach

A-1. Definitions of external instruments

We checked the robustness of our findings against the inclusion of external instruments to address a potential weak instrument problem. Here, we used two external instruments: the average daily steps within the same school district except for a participant him/herself (IV1) and the average daily steps of participants who lived in nearby school districts spatially weighted by inverse distance (IV2). They measure the walkability of the neighbouring environment of the participants. If the neighbouring environment of a participant is easy to walk, all the participants in those areas will walk. To measure the walkability of the participant's neighbourhood, two instruments were made from other participants' daily steps. Capturing the walkability of the neighbouring environment, these variables were correlated with the average daily steps of a participant but not with their medical expenditure. For the first instrument, we used the average daily steps of participants within the same school area in the same year calculated as follows:

$$Average\ daily\ steps\ of\ participants\ within\ the\ same\ school\ area\ for\ participant\ i$$
$$= \frac{Sum\ of\ average\ daily\ steps\ of\ participants\ within\ the\ same\ school\ area\ in\ the\ same\ year}{number\ of\ participants\ in\ the\ same\ school\ area\ in\ the\ same\ year}$$

where the average daily steps of the participant i was excluded from both the denominator and numerator.

Second, the weighted average of daily steps of participants in near school areas was calculated. The ith weight $w_{ij} = 1/d_{ij}$, which was used to calculate the weighted average of the ith participants, was obtained as the inverse of the distance between the location of an elementary school of the ith participant and the location of elementary schools where the jth participant lived, and w_{ij} $(j = 1, \cdots, n)$ were normalised to add up to one. Coordinates of elementary schools were used to measure the distances between participants since the home address of all participants was not available. Additionally, other weights were calculated for robustness checks: the weights were based on inverse distances limited up to a certain distance (i.e. 2 km, 4 km, and 6 km), and the weights were one ($w_{ij} = 1$) if the neighbouring participants lived within the distance. As we obtained similar results for both the definitions of a spatially weighted average of daily steps of participants, we only reported the result using the former definition of a spatially weights.

A-2. Results

Table A-1 and Table A-2 represent the estimation results of fixed-effects OLS regression and system GMM using external instruments for outpatient and inpatient costs, respectively. The validities of models by system GMM were confirmed by the Hansen test and Arellano-Bond test for AR(2). The effects of daily steps on outpatient costs but not on inpatient costs, and in women but not in men were significant in consistent with our main analyses. Furthermore, those effects were similar magnitude for the whole sample (main analysis, β: -16.26, SE: 6.51 vs IV 1, β: -16.44, SE: 6.56 vs IV 2, β: -16.07,

SE: 6.54) and for women (main analysis, β: -23.18, SE: 7.84 vs IV 1, β: -22.62, SE: 7.88 vs IV 2, β: -23.30, SE: 7.84). Thus, we confirmed the robustness of our findings against the potential weak instrument issue.

Table A-1. Effects of daily step on healthcare costs: Outpatient services (External IV)

	1. System GMM External IV: average daily steps within same school district			2.System GMM External IV: spatial weight of inverse average daily steps of nearby school district		
Variable	Whole	Male	Female	Whole	Male	Female
Daily steps	-16.44**	-11.15	-22.62***	-16.07**	-3.42	-23.30***
	(6.56)	(10.56)	(7.88)	(6.54)	(8.47)	(7.84)
Daily steps: long run effect	-28.56**	-19.71	-41.28***	-28.18**	-6.58	-42.58***
	(11.47)	(18.47)	(14.37)	(11.61)	(16.35)	(14.40)
Outpatient costs (JPY) (t-1)	0.42***	0.43***	0.45***	0.43***	0.48***	0.45***
	(0.05)	(0.13)	(0.07)	(0.05)	(0.14)	(0.07)
Age	4,852***	2,869	5,026***	4,787***	2,829	5,056***
	(877.2)	(1,920)	(1,071)	(884.7)	(1,952)	(1,079)
Male (=1)	31,068***			29,551***		
	(11,462)			(11,398)		
Individual fixed effects	Yes	Yes	Yes	Yes	Yes	Yes
Year fixed effects	Yes	Yes	Yes	Yes	Yes	Yes
City fixed effects	Yes	Yes	Yes	Yes	Yes	Yes
Constant	-49,047	74,695	-30,343	-48,922	1,976	-27,592
	(66,966)	(151,725)	(75,347)	(66,921)	(140,912)	(75,342)
Number of observations	2,419	560	1,859	2,430	565	1,865
Number of individuals	1,072	258	814	1,074	259	815
1st stage specification tests						
Arellano-Bond: AR(2) z	-0.497	0.979	-1.066	-0.488	1.037	-1.059
	[0.619]	[0.328]	[0.286]	[0.625]	[0.300]	[0.290]
Hansen J	15.60	15.35	15.64	16.27	16.27	15.41
	[0.481]	[0.499]	[0.479]	[0.434]	[0.435]	[0.494]
Number of Instruments	27	26	26	27	26	26

a. Robust standard errors in parentheses. *** p<0.01, ** p<0.05; P-values in brackets

b. The long-run effects of daily step on healthcare costs are calculated by the coefficient of daily steps divided by 1 minus the lagged coefficient of the dependent variable.

Table A-2. Effects of daily step on healthcare costs: Inpatient services (External IV)

	1. System GMM External IV: average daily steps within same school district			2. System GMM External IV: spatial weight of inverse average daily steps of nearby school district		
Variable	Whole	Male	Female	Whole	Male	Female
Daily steps	2.70	12.05	1.96	0.57	-0.01	1.91
	(13.50)	(29.63)	(9.66)	(13.40)	(23.83)	(9.64)
Daily steps: long run effect	3.08	14.01	2.26	0.65	-0.01	2.19
	(15.51)	(35.09)	(11.15)	(15.22)	(28.93)	(11.10)
Inpatient costs (JPY) (t-1)	0.12	0.14	0.13**	0.12	0.18	0.13**
	(0.07)	(0.11)	(0.06)	(0.07)	(0.11)	(0.06)
Age	2,335***	4,150	1,430	2,418***	4,856**	1,424
	(778.4)	(2,781)	(809.4)	(774.0)	(2,255)	(801.5)
Male (=1)	28,101			31,796**		
	(14,993)			(15,466)		
Individual fixed effects	Yes	Yes	Yes	Yes	Yes	Yes
Year fixed effects	Yes	Yes	Yes	Yes	Yes	Yes
City fixed effects	Yes	Yes	Yes	Yes	Yes	Yes
Constant	-140,879	-295,663	-85,830	-130,970	-267,410	-85,659
	(104,403)	(355,489)	(83,148)	(101,603)	(272,546)	(84,728)
Number of observations	2,423	560	1,863	2,434	565	1,869
Number of individuals	1,067	255	812	1,069	256	813
1st stage specification tests						
Arellano-Bond: AR(2) z	0.764	0.645	0.268	0.675	0.217	0.279
	[0.445]	[0.519]	[0.789]	[0.500]	[0.829]	[0.780]
Hansen J	17.06	14.70	15.04	16.16	15.12	15.03
	[0.381]	[0.546]	[0.522]	[0.442]	[0.516]	[0.522]
Number of Instruments	27	26	26	27	26	26

a. Robust standard errors in parentheses. *** p<0.01, ** p<0.05; P-values in brackets
b. The long-run effects of daily step on healthcare costs are calculated by the coefficient of daily steps divided by 1 minus the lagged coefficient of the dependent variable.

B. Multiple imputation

B-1. Multiple imputation

In our main analyses, although we controlled for healthcare costs in previous year as a proxy of one's health status, other health-related variables were not included to control for confounders due to the data restriction. For the robustness check, we utilised the result of health check-ups performed every year under the scheme of national health insurance. Among the results, we adopted the information on metabolic syndrome, which is considered to be one of the factors that accounted for increasing healthcare costs[1], as a control variable in the panel data analysis with fixed-effects. Metabolic syndrome is ascertained based on the Japanese criteria[2] for an individual with their waist circumstance over 85 cm for men and 90 cm for women, and two or more of three risk factors: (1) 150 mg/dl ≦triglyceride and/or high-density lipoprotein cholesterol <40 mg/dl; (2) 85 mmHg ≦diastolic blood pressure and/or 130 mmHg ≦ systolic blood pressure; (3) 110 mg/d ≦fasting blood glucose.

However, the information on metabolic syndrome is available only for those who participated in health check-ups, thereby the decreasing sample size will increase standard errors, and the selection bias becomes problematic if we exclude those who do not take check-ups. Therefore, we imputed the data regarding metabolic syndrome assessment in the e-wellness participants by the following approach.

B-2. Statistical Analysis

We used the multiple imputation procedure in Stata software to impute the missing data on metabolic syndrome. The multiple imputation method consists of three phases: the imputation phase, the analysis phase, and the pooling phase.

In the imputation phase, we created 50 imputed data sets. The imputation model was analysed using a logistic regression model that explained the metabolic syndrome dummy by age, sex, medical expenses for outpatient/inpatient services, annual average counts of daily steps, residential city, and year dummy variables.

In the analysis phase, the metabolic syndrome dummy was added as a control variable in the fixed-effects linear regression model in the main text, and regression analysis was performed for each of the 50 datasets. In the pooling phase, the results from 50 datasets were integrated. We analysed according to different assumptions about the missing data mechanisms: missing completely at random (MCAR) and missing at random (MAR).

B-3. Result

Table B-1 shows the comparison between participants with/without the information on metabolic syndrome. Differences in the key variables for our analyses (i.e. outpatient/inpatient costs, age, and step counts) were found to be significant between the two groups: participants with the information have lower healthcare costs for outpatient and inpatient services, were younger, and

walked more than those without a variable in metabolic syndrome. While this did not differ between men and women, daily steps were significantly different between men and women as shown in Table 1. Therefore, we controlled for sex in the imputation phase.

Table B-2 shows the results of multiple imputation analyses by the fixed-effects linear model of the association between step counts and healthcare costs, comparing the results based on the different assumptions about the missing data mechanisms. In all models, the association was found to be significant: step counts were negatively associated with healthcare costs. Furthermore, the coefficients of daily steps were similar from our main analyses.

We compared the results of analyses between models with/without metabolic syndrome dummy variables. The small differences in coefficients between these models suggested that our sample, namely, the participants in the e-wellness project were not a special group. As shown in the table, the results for both groups were similar. Thus, we confirmed that our main findings were not severely biased owing to selection bias that the participants were healthy elites. Thus, we confirmed that unmeasured confounders of health condition do not have serious effects of daily steps on healthcare costs.

Table B-1. Descriptive statistics: The Difference of means between complete and incomplete data

	(i) Complete Data [a]		(ii)Incomplete Data [b]		
Variable [c]	Mean	S. D.[d]	Mean	S. D.	(i)-(ii)e
Inpatient costs (JPY) [f, g]	38,106	207,270	60,394	279,369	##
Outpatient costs (JPY) [f, g]	233,003	195,855	265,126	231,203	###
Daily steps (annual average)	6,980	2,914	6,521	2,974	***
Age	65.65	5.363	66.46	6.28	***
Male (=1)	0.24	0.424	0.23	0.423	
City A(=1)	0.36	0.48	0.26	0.436	###
City B(=1)	0.45	0.497	0.41	0.492	
City C(=1)	0.20	0. .397	0.33	0.472	***
YEAR 2009(=1)	0.17	0.371	0.11	0.309	###
YEAR 2010(=1)	0.18	0.388	0.21	0.406	
YEAR 2011(=1)	0.20	0.399	0.22	0.411	
YEAR 2012(=1)	0.23	0.418	0.23	0.424	
YEAR 2013(=1)	0.23	0.419	0.24	0.424	
N. of observations	3,041		943		

a. Complete Data: participants who took health check-ups and with the information on metabolic syndrome diagnosis.

b. Incomplete Data: participants who did not take health check-ups and without the information on metabolic syndrome diagnosis.

c. (=1) indicates dummy variable.

d. S.D. stands for standard deviation.

e. ***, ** indicate the positive results of a t-test of equal means between two samples at the 1% and 5% significance levels, respectively. ### and ## indicate the negative results of the t-test at the 1% and 5% significance levels, respectively. We use Welch's method to test the difference of averages under the hypothesis of heteroscedasticity; standard deviation in parentheses.

f. Number of observations of inpatient cost is different: complete data n=3,050, incomplete data n=941.

g. The unit of currency is 1JPY (≒0.007 GBD).

Table B-2. Multiple imputation analyses by the fixed-effects linear model: step counts and healthcare costs [a, b]

		Inpatient cost	Outpatient cost
	Missing assumption	β	β
Without metabolic syndrome		-17.25***	-14.00***
		(6.03)	(3.08)
With metabolic syndrome	MCAR [c]	-16.33**	-15.49***
		(7.84)	(3.18)
	MAR [d]	-16.38***	-13.95***
		(6.13)	(3.08)

a. *** p<0.01, ** p<0.05; Robust standard errors in parentheses
b. Adjusted for age, residential city, and year.
c. MCAR: missing completely at random. We perform listwise deletion.
d. MAR: missing at random.

C. Data augmentation of personal income from panel survey

In Appendix B, we confirmed that an uncontrolled health variable in our main analyses did not severely affect our main findings. Moreover, our data set lacked the information on economic factors, such as income or assets of the participants, which may cause omitted variable bias. Therefore, we further conducted robustness test by augmenting income using the following method. Specifically, augmented income was drawn as following two steps based on Bayesian techniques[3]:

Posterior-step: We write augmented income of the gth sampling as

$$\mathrm{y}_{\mathrm{n}}^{*(\mathrm{g})} = (y_1, \cdots, y_r, y_{r+1}^{*(g)}, \cdots, y_n^{*(g)})'.$$

Here, $(y_1, \cdots, y_r)$ denotes the logarithms of incomes of respondents of a survey and $y^* = (y_{r+1}^*, \cdots, y_n^*)$ represents the logarithms of potential incomes of participants. X is a corresponding matrix of covariate matrix: $X = \left(X_1, \cdots, X_r, X_{r+1,} \cdots X_n\right)$.

Prior distribution is as follows:

$$\beta \sim \mathrm{N}(\underline{\beta}, \underline{V}),\ \ \mathrm{h} \sim \mathrm{G}(\frac{\underline{\nu}}{2}, \frac{2}{\underline{\nu s^2}})$$

Conditional posterior distributions are as follows:

$$\mathrm{h}^{*(\mathrm{g})}|\mathrm{y}_{\mathrm{n}}^{*(\mathrm{g})} \sim \mathrm{G}\left(\frac{v_1}{2}, \frac{2}{v_1 s_{12} + \underline{\nu s^2}}\right) = \left(\frac{2}{\mathrm{v}_1 s_{12}}\right)^{-\frac{v_1}{2}} \Gamma\left(\frac{v_1}{2}\right)^{-1} h^{\frac{v_1}{2}-1} exp\left(-\frac{h}{2}\frac{2}{\mathrm{v}_1 s_{12} + \underline{\nu s^2}}\right)$$

$$\beta^{*(\mathrm{g})}|y_n^{*(g)}, \sigma^{*(g)2} \sim \mathrm{N}(\hat{\beta}^{(g-1)},\ (h^{*(g-1)})^{-1}\ (\mathrm{X}_{\mathrm{n}}' X_n)^{-1})$$

$$\sigma^{*(g)2} = (y_n - X_n\hat{\beta}^{(g-1)})'(y_n - X_n\hat{\beta}^{(g-1)})/v_1$$

$$\mathrm{h}^{*(\mathrm{g})} = 1/\sigma^{*(g)2} = v_1/(y_n - X_n\hat{\beta}^{(g-1)})'(y_n - X_n\hat{\beta}^{(g-1)})$$

$$\mathrm{v}_1 = n + \underline{\nu}$$

$$\hat{\beta}^{(g)} = \left(h^{*(g)}X_n'X_n + \underline{V}^{-1}\right)^{-1}(\underline{V}^{-1}\underline{\beta} + h^{*(g)}X_n'y_n^{*(g)})$$

We sample β^* and h^* by Gibbs sampling based on those distributions.

Imputation-step: We imputed the potential income of the participants form the following distribution:

$$\mathrm{y}_\mathrm{n}^{*(\mathrm{g})} \sim N(X\upbeta^{*(\mathrm{g})}, h^{*-1(g)})$$

We obtained the logarithms of income $(y_1, \cdots, y_r)$ and covariates $(X_1, \cdots, X_r)$ from the Japan Household Panel Survey (JHPS) 2009-2015, a national representative household survey conducted every year in Japan. The data of $(X_{r+1}, \cdots, X_n)$ correspond to the covariates of the e-wellness participants. We used earnings from employment (including earnings from self-employment) and annuities for y. For covariates X, we used age, age-squared, city sizes fixed-effects (12 quartiles), and prefecture fixed-effects (47 prefectures). We sampled parameters from the posterior distribution by Gibbs sampling using conditional posterior distributions. We generated 11,000 observations, of which 1,000 were discarded. We used OLS estimator b_{OLS} by regressing y_r on X_r for the parameter of prior distribution $\beta \sim N(b_{OLS}, V)$. For the diagonal elements of V, 0.01^2 were set. For the parameter of precision, h, $\underline{v}$=5 and $\underline{s}^2 = 1$ were used.

Table C-1 presents summary statistics of posterior distribution of $\hat{\beta}_n$ and h. Table C-2 shows the summary statistics of augmented incomes. Table C-3 and C-4 depict results obtained by the models to explain outpatient/inpatient costs after controlling augmented incomes. We confirmed that estimates

of daily steps of fixed-effects and system GMM models remain unchanged from Table 2 and 3. Thus, we confirmed that our main findings are robust against the models including income.

Table C-1: Summary statistics of posterior distribution

Dependent variable	Earnings from employment (10 thousand yen)			Annuities (10 thousand yen)		
Variable	Mean	S.D.	C.D.	Mean	S.D.	C.D.
Age	0.342	4.13E-06	-0.320	-0.151	4.15E-06	1.067
Age squared	-0.004	5.10E-08	0.433	0.003	5.09E-08	-1.122
Male (=1)	1.402	4.56E-05	1.524	0.195	4.62E-05	1.987
City size: 12 quartiles		Yes			Yes	
Survey year fixed-effects		Yes			Yes	
Prefecture fixed-effects		Yes			Yes	
Precision: h	43962.597	337.128	-0.868	43960.921	335.244	0.440
N		34,541			34,541	
N: JHPS		30,538			30,538	
N: SWC		4,003			4,003	

Note: Observation periods: 2009-2013. Earnings from employment includes earnings from self-employment. We generated 11,000 observations, of which 1,000 are discarded. We generated 11,000 observations, of which 1,000 are discarded. We used OLS estimator b_{OLS} for the parameter of prior distribution $\beta \sim N(b_{OLS}, V)$. For the diagonal elements of V, 0.01^2 are set. For the parameter of precision, h, $\underline{\nu}$=5 and $\underline{s}^2 = 1$ are used. C.D. stands for the Geweke (1992)'s convergence diagnostic[4].

Table C-2: Summary statistics of augmented incomes

Unit: 10 thousand JPY

	Earnings from employment			Annuities		
	Female	Male	Whole	Female	Male	Whole
40s						
Mean	60.396962	222.3467	104.35475	0.872349	0.9168932	0.8844396
S.D.	32.094849	116.82211	97.818511	0.2053972	0.286346	0.2288206
N	51	19	70	51	19	70
50s						
Mean	30.114121	100.48848	38.575554	3.167479	3.6494666	3.2254306
S.D.	18.178527	56.947978	34.605782	1.6775312	1.3486177	1.6472343
N	300	41	341	300	41	341
60s						
Mean	9.9555784	34.429655	15.200023	15.695438	20.341472	16.691017
S.D.	6.8233991	22.280604	15.610553	8.397164	9.6585626	8.8878382
N	1991	543	2534	1991	543	2534
70s						
Mean	2.6317285	10.550371	5.1839563	63.016285	78.992434	68.165498
S.D.	1.7533614	6.0345134	5.2445492	25.329315	32.297858	28.739235
N	717	341	1058	717	341	1058
Total						
Mean	11.056877	32.455078	16.103068	25.311222	40.411958	28.872325
S.D.	13.18461	42.426937	25.288617	25.455479	35.919257	28.987054
N	3059	944	4003	3059	944	4003

Note: Observation periods: 2009-2013; S.D. stands for standard deviation.

Table C-3: Robustness checks of effects of daily step on healthcare costs controlling augmented incomes: Outpatient costs

	1. Fixed-effects OLS			2. System GMM		
Variables	Whole	Male	Female	Whole	Male	Female
Daily steps	-14.22***	-9.449**	-17.12***	-16.79**	-8.218	-22.41***
	(3.026)	(4.758)	(3.857)	(6.543)	(10.04)	(7.792)
Daily steps:				-29.12	-14.69	-40.96
Long run effects				(11.520)	(18.020)	(14.310)
Outpatient costs				0.423***	0.441***	0.453***
(JPY) (t-1)				(0.0531)	(0.115)	(0.0672)
Age	21,050***	22,119	22,811***	7,600***	5,740	11,604***
	(5,848)	(13,567)	(6,554)	(1,548)	(6,582)	(2,658)
Male (=1)				27,387**		
				(13,708)		
Earning income from	103.9	-106.2	960.6	301.9	143.1	1,764**
employment (JPY)	(377.9)	(476.0)	(724.5)	(254.9)	(642.7)	(702.2)
Annuities (JPY)	-1,044***	-2,005***	-765.1*	-406.5**	-336.4	-750.9**
	(350.7)	(632.8)	(437.8)	(205.7)	(419.4)	(314.3)
Individual fixed effects	Yes	Yes	Yes	Yes	Yes	Yes
Year fixed effects	Yes	Yes	Yes	Yes	Yes	Yes
City fixed effects	Yes	Yes	Yes	Yes	Yes	Yes
Constant	-1.02e+06***	-1.03e+06	-1.15e+06***	-220,853**	-133,807	-466,745***
	(391,979)	(933,078)	(434,603)	(105,729)	(504,877)	(171,733)
Number of observations	3,984	938	3,046	2,430	565	1,865
Number of individuals	1,532	368	1,164	1,074	259	815
1st stage specification tests						
Arellano-Bond: AR(2) z				-0.503	1.017	-1.074
p-values				[0.615]	[0.309]	[0.283]
Hansen J				15.12	13.54	15.94
p-values				0.443	0.561	0.386
Number of instruments				28	27	27

a. Robust standard errors in parentheses. *** p<0.01, ** p<0.05. P-values in brackets

b. The long-run effects of daily step on healthcare costs are calculated by the coefficient of daily steps divided by 1 minus the lagged coefficient of the dependent variable.

c. Abbreviations: OLS＝Ordinary Least Squares, GMM=Generalized Method of Moments

Table C-4: Robustness checks of effects of daily step on healthcare costs controlling augmented incomes: Inpatient costs

	1. Fixed-effects OLS			2. System GMM		
Variables	Whole	Male	Female	Whole	Male	Female
Daily steps	-17.10***	-13.66	-18.53***	0.705	-1.336	1.801
	(6.010)	(12.35)	(6.364)	(13.54)	(23.49)	(9.882)
Daily steps:				0.803	-1.570	2.072
Long run effects				(15.45)	(27.54)	(11.40)
Inpatient costs (JPY)				0.121*	0.149	0.131**
(t-1)				(0.0687)	(0.105)	(0.0603)
Age	-5,741	64,345*	-23,835**	20.51	13,740	-1,201
	(10,656)	(33,330)	(9,863)	(1,221)	(13,213)	(1,931)
Male (=1)				35,894*		
				(19,781)		
Earning income	-946.7**	-300.6	-1,333	-328.6	1,196	-594.5
from employment (JPY)	(449.7)	(520.0)	(886.6)	(251.3)	(1,346)	(524.4)
Annuities (JPY)	776.3	810.2	209.1	343.4	-262.2	379.9
	(604.5)	(1,097)	(647.5)	(254.1)	(667.0)	(381.8)
Individual fixed effects	Yes	Yes	Yes	Yes	Yes	Yes
Year fixed effects	Yes	Yes	Yes	Yes	Yes	Yes
City fixed effects	Yes	Yes	Yes	Yes	Yes	Yes
Constant	545,686	-<br>4.218e+06*	1.762e+06***	21,370	-878,031	85,678
	(716,101)	(2.271e+06)	(664,445)	(119,293)	(1.030e+06)	(127,795)
Number of observations	3,991	939	3,052	2,434	565	1,869
Number of individuals	1,534	368	1,166	1,069	256	813
1st stage specification tests						
Arellano-Bond: AR(2) z				0.664	-0.0118	0.297
p-values				0.507	0.991	0.766
Hansen J				15.50	13.23	14.62
p-values				0.416	0.585	0.479
Number of instruments				28	27	27

a. Robust standard errors in parentheses. *** $p<0.01$, ** $p<0.05$. P-values in brackets

b. The long-run effects of daily step on healthcare costs are calculated by the coefficient of daily steps divided by 1 minus the lagged coefficient of the dependent variable.

c. Abbreviations: OLS＝Ordinary Least Squares, GMM=Generalized Method of Moments

References for Supplementary Material

1 Fu, T., Wen, T., Yeh, P. & Chang, H. Costs of metabolic syndrome-related diseases induced by obesity in Taiwan. *Obes. Rev.* **9 Suppl 1**, 68-73, doi:10.1111/j.1467-789X.2007.00441.x (2008).

2 Matsuzawa, Y. Metabolic syndrome--definition and diagnostic criteria in Japan. *J Atheroscler Thromb* **12**, 301, doi:10.5551/jat.12.301 (2005).

3 Kim, J. K. & Shao, J. *Statistical methods for handling incomplete data*. (Chapman and Hall/CRC, 2013).

4 Geweke, J. in *Bayesian Statistics 4* (eds J. M. Bernardo, James O. Berger, J. O. Dawid, & A. F. M. Smith) (Oxford University Press, 1992).
